# *Sleeping Beauty* Transposition of Chimeric Antigen Receptors Targeting Receptor Tyrosine Kinase-Like Orphan Receptor-1 (ROR1) into Diverse Memory T-Cell Populations

**DOI:** 10.1371/journal.pone.0128151

**Published:** 2015-06-01

**Authors:** Drew C. Deniger, Jianqiang Yu, M. Helen Huls, Matthew J. Figliola, Tiejuan Mi, Sourindra N. Maiti, George F. Widhopf, Lenka V. Hurton, Radhika Thokala, Harjeet Singh, Simon Olivares, Richard E. Champlin, William G. Wierda, Thomas J. Kipps, Laurence J. N. Cooper

**Affiliations:** 1 Pediatrics, Children’s Cancer Hospital, University of Texas MD Anderson Cancer Center, Houston, Texas, United States of America; 2 University of Texas Graduate School of Biomedical Sciences at Houston, Houston, Texas, United States of America; 3 Medicine, Moores Cancer Center, University of California San Diego, San Diego, California, United States of America; 4 Stem Cell Transplantation and Cellular Therapy, University of Texas MD Anderson Cancer Center, Houston, Texas, United States of America; 5 Leukemia, University of Texas MD Anderson Cancer Center, Houston, Texas, United States of America; Jackson Laboratory, UNITED STATES

## Abstract

T cells modified with chimeric antigen receptors (CARs) targeting CD19 demonstrated clinical activity against some B-cell malignancies. However, this is often accompanied by a loss of normal CD19^+^ B cells and humoral immunity. Receptor tyrosine kinase-like orphan receptor-1 (ROR1) is expressed on sub-populations of B-cell malignancies and solid tumors, but not by healthy B cells or normal post-partum tissues. Thus, adoptive transfer of T cells specific for ROR1 has potential to eliminate tumor cells and spare healthy tissues. To test this hypothesis, we developed CARs targeting ROR1 in order to generate T cells specific for malignant cells. Two *Sleeping Beauty* transposons were constructed with 2^nd^ generation ROR1-specific CARs signaling through CD3ζ and either CD28 (designated ROR1RCD28) or CD137 (designated ROR1RCD137) and were introduced into T cells. We selected for T cells expressing CAR through co-culture with γ-irradiated activating and propagating cells (AaPC), which co-expressed ROR1 and co-stimulatory molecules. Numeric expansion over one month of co-culture on AaPC in presence of soluble interleukin (IL)-2 and IL-21 occurred and resulted in a diverse memory phenotype of CAR^+^ T cells as measured by non-enzymatic digital array (NanoString) and multi-panel flow cytometry. Such T cells produced interferon-γ and had specific cytotoxic activity against ROR1^+^ tumors. Moreover, such cells could eliminate ROR1^+^ tumor xenografts, especially T cells expressing ROR1RCD137. Clinical trials will investigate the ability of ROR1-specific CAR^+^ T cells to specifically eliminate tumor cells while maintaining normal B-cell repertoire.

## Introduction

T cells can be rendered specific for tumor-associated antigens (TAAs) independent of their endogenous T-cell receptor (TCR) via gene transfer of chimeric antigen receptors (CARs) [1]. CARs are constructed from the genes encoding a single-chain variable fragment (scFv) of a TAA-specific monoclonal antibody (mAb), extracellular hinge or scaffold with transmembrane domain, and portions of CD3ζ and CD28 or CD137 (4-1BB) endodomains. Introduction of this chimeric gene generates T cells that proliferate, produce cytokines, and direct cytolysis of tumor cells in a TAA-dependent manner [2]. Infusion of T cells expressing CAR specific for CD19 with either CD3ζ /CD28 or CD3ζ /CD137 can induce complete tumor regressions in subsets of patients with B-lineage lymphomas, acute lymphoblastic leukemia (B-ALL), or chronic lymphocytic leukemia (CLL) [3–10]. In addition to the structure of the CAR, the subset of T cells that serves as a template for bioengineering can impact the anti-tumor effect. For instance, murine immunotherapy models have demonstrated that less differentiated T cells, *i*.*e*., naïve (T_N_), stem-cell memory (T_SCM_), or central memory (T_CM_) T cells, can clear tumor cells more effectively than more differentiated T cells, *i*.*e*., effector memory (T_EM_) or terminally-differentiated effector memory RA (T_EMRA_) T cells [11, 12]. Thus, the specificity of the T cell and the underlying memory phenotype can be important predictors for therapeutic efficacy.

T cells that express CARs specific for CD19 cannot distinguish between neoplastic or normal CD19-bearing B cells. Indeed, the initial clinical data has demonstrated that most patients benefiting from an anti-tumor response have concomitant B-cell depletion. Such B-cell aplasia requires that the patient receive timely intravenous infusions of normal immunoglobulin to alleviate the threat for opportunistic infections [13]. Nevertheless, such treatment cannot eliminate the risk for serious infection, as one recipient of CD19-specific CAR^+^ T cells died due to opportunistic infection [14]. Thus, targeting a TAA, which is not expressed on normal B cells or other adult tissues, would mitigate the risk for B-cell depletion and potentially improve outcome.

One TAA that may serve as an alternative to CD19-directed T-cell therapy is receptor tyrosine kinase-like orphan receptor-1 (ROR1). ROR1 is principally expressed during embryonic development but its expression attenuates during fetal development and is negligible at term [15]. However, ROR1 is aberrantly expressed by some B-cell malignancies, *e*.*g*., lymphomas, CLL, and t(1;19) B-ALL, and by many solid-tumor malignancies, *e*.*g*., adrenal, bladder, breast, colon, lung, pancreas, prostate, ovary, skin, testes, uterus, and neuroblastoma [16–21]. ROR1 has also been discovered on ovarian cancer stem cells yielding the potential to eliminate tumor initiating cells by targeting ROR1 [22]. Monoclonal antibodies specific for ROR1 have reacted with some bone marrow adipocytes [23] and hematogones [24] (B-cell precursors dispensable for maintaining peripheral B-cell repertoire), but not on other normal tissues [18, 20]. Non-human primates treated with autologous ROR1-specific T cells had no toxicity from treatment suggesting that targeting ROR1 is a safe approach for humans [25]. Thus, it appears T cells that express CARs specific for ROR1 would not deplete B-cells, but rather target neoplastic B cells or solid tumors.

Clinical trials have not yet evaluated the efficacy or safety of targeting ROR1 using adoptive immunotherapy. In preparation for the human application of genetically modified T cells, we evaluated two 2^nd^ generation CAR species specific for ROR1. Our approach is based on the methodology employed in our first-in-human clinical trials using CD19-specific T cells generated by synchronous electro-transfer of CD19-specific CAR as a *Sleeping Beauty* (SB) transposon and a hyperactive SB transposase [26, 27]. Following transfection the T cells are co-cultured with irradiated activating and propagating cells (AaPC), which select for T cells that have stable expression of the CAR through direct interactions with AaPC bearing its cognate antigen, *e*.*g*., CD19 [28–31]. In this study we generated AaPC that express ROR1 and developed SB transposons to express CARs specific for ROR1 that signal via chimeric CD3ζ/CD28 (designated ROR1RCD28) or chimeric CD3ζ/CD137 (designated ROR1RCD137). We show that T cells expressing either CAR can proliferate in response to cells bearing ROR1 and specifically kill ROR1^+^ tumor cells. We found that ROR1RCD137^+^ T cells had more effective anti-tumor activity than ROR1RCD28^+^ T cells in immune-deficient mice engrafted with ROR1^+^ tumor cells. A phase I clinical trial (NCT02194374) is open for the infusion of autologous ROR1RCD137 CAR^+^ T cells in patients with CLL.

## Materials and Methods

### Ethics statement

All human samples acquired for this study were obtained after written informed consent was granted in accordance with protocols established and approved by the MD Anderson Cancer Center Internal Review Board (IRB). The identities of all samples were kept private. Animals treated in this study were handled in accordance with the strict guidelines established by the MD Anderson Cancer Center Institutional Animal Care and Use Committee (IACUC), which specifically approved this study (Protocol#03-06-04333). Mice were housed in pathogen-free conditions and were monitored daily for welfare-related assessments in accordance to IACUC guidelines. Moribund mice, *e*.*g*., ruffled fur, hunched posture, tumor size, were humanely euthanized with inhaled CO_2_ as per direction of IACUC guidelines. No mice died without euthanasia. All efforts were made to minimize animal suffering and inhaled isoflurane was administered for anesthesia as required.

### Chimeric antigen receptors

Cloning of second generation CD19-specific CARs signaling through CD3ζ and CD28 (CD19RCD28) or CD137 (CD19RCD137) have been previously described [32, 33]. Heavy and light chain immunoglobulin sequences from the 4A5 mAb hybridoma were codon optimized and synthesized *de novo* (GeneArt; Invitrogen, Grand Island, NY) to create the “ROR1R” nucleotide sequence of (i) murine IgGκ signal peptide, (ii) V_L_, (iii) Whitlow linker (GSTSGSGKPGSGEGSTKG), (iv) V_H_, and (v) the first 73 amino acids of a modified human IgG4 stalk. ROR1R was amplified by PCR with ROR1RCoOpF (GCTAGCCGCCACCATGGGCTGGTCCTGCATC) and ROR1Rrev (GCTCCTCCCGGGGCTTTGTCTTGGC) primers then sub-cloned into pCR4-TOPO with TOPO TA Cloning Kit (Invitrogen) to generate ROR1R(CoOp)/pCR4-TOPO and sequence was verified with T7 and T13-0 primers (DNA Sequencing Core, MDACC). ROR1R(CoOp)/pCR4-TOPO and CD19RCD28mZ(CoOp)/pEK plasmids were digested with *NheI* and *SmaI* and ligated to generate ROR1RCD28mZ(CoOp)/pEK. The ROR1-specific CAR was then transferred into a SB transposon by digestion of CD19RCD28mZ(CoOp)/pSBSO-MCS and ROR1RCD28mZ(CoOp)/pEK with *NheI* and *SpeI* to generate ROR1RCD28mZ(CoOp)/pSBSO-MCS. The final ROR1RCD28 SB transposon plasmid was constructed by digesting CD19RCD28mZ(CoOp)/pSBSO-SIM with *NheI*, *XmaI*, and *Antarctic Phosphatase* and ROR1RCD28mZ(CoOp)/pSBSO-MCS with *NheI*, *XmnI*, and *XmaI* to generate ROR1RCD28/pSBSO-SIM plasmid. Similarly, the final ROR1RCD137 transposon plasmid was constructed by digesting CD19R-CD28Tm-41BBCyt-Z(CoOp)/pSBSO-FRA with *NheI*, *XmaI*, and *Antarctic Phosphatase* and ROR1RCD28mZ(CoOp)/pSBSO-MCS with *NheI*, *XmnI*, and *XmaI* to generate ROR1RCD137/pSBSO-FRA plasmid. Identities of final ROR1R plasmids were distinguished from one another with *BsrGI* and from CD19R plasmids by *PmlI* (not present). The entire sequence of both plasmids was verified by Sanger Sequencing (DNA Sequencing Core, MDACC).

### Tumor cell tissue culture

EL4 cell line was acquired from American Type Culture Collection (Manassas, VA; cat# ATCC TIB-39). NALM-6 cell line was purchased from Deutsche Sammlung von Mikroorganismen und Zellkulturen (Germany; cat# ACC-128). Kasumi-2 was a gift from Jeffrey Tyner (Oregon Health & Science University) [34]. Clone#9 AaPC (previously referred to as artificial antigen presenting cells; aAPC) was generated though enforced co-expression of truncated CD19, CD64, CD86, and CD137L on K-562 cells genetically modified with lentiviral vectors and was a gift from Carl June (University of Pennsylvania) [35]. This AaPC was further modified using SB system to co-express IL15/IL15Rα fusion protein (designated membrane-bound IL-15; mIL15) and sub-cloned to generate clone#27. Clone#27 was then genetically modified using SB system to express ROR1, and single-cell clones were isolated by FACS based on co-staining of ROR1, CD137L, and IL15 during the sort. The clone#1 AaPC uniformly and stably co-expressed CD19, CD32, CD64, CD86, CD137L, mIL15, and ROR1. These cell lines were maintained in complete media (RPMI, 10% FBS (Hyclone, Logan, UT), and 1x Glutamax-100). Identities of all cell lines were confirmed by STR DNA Fingerprinting at MDACC’s Cancer Center Support Grant (CCSG) supported facility “Characterized Cell Line Core.” All tumor cells were free of mycoplasma and other microbial pathogens.

### Numeric expansion of ROR1-specific CAR^+^ T cells

CAR^+^ T cells were propagated based on modifying standard operating protocols as previously described [28, 32]. Cryopreserved PBMC, obtained from healthy donors after informed consent, were thawed the day of the electroporation (designated day 0) and rested for 2 hours at 37°C. PBMC for electroporation were spun at 200g for 10 minutes and 2x10^7^ cells were mixed with supercoiled DNA plasmids (5 μg SB11 transposase and 15 μg SB transposon) in Human T cell Nucleofector Solution (cat#VPA-1002, Lonza), added to a cuvette, and electroporated on the U-014 program of Amaxa Nucleofector II (Lonza). Electroporated cells were transferred to a 6-well plate containing phenol-free RPMI, 20% FBS, and 1x Glutamax-100. The following day, electroporated T cells were phenotyped and stimulated with AaPC clone#1 (irradiated using cesium source to 100 Gy) at 1:1 ratio of clone#1 to ROR1-specific CAR^+^ T cells. Each co-culture was supplemented with IL-21 (cat# AF20021; Peprotech, Rocky Hill, NJ; 30 ng/mL) starting at initiation of culture and every 2–3 days thereafter and with IL-2 (Aldesleukin; Novartis, Switzerland; 50 IU/mL) added every 2–3 days starting at the second stimulation. CAR expression was evaluated weekly to determine the number of AaPC to add to co-cultures every 7 days. If contaminating NK cells reached >10% of the total population, they were depleted from co-cultures with paramagnetic CD56 microbeads (cat#130-050-401, Miltenyi Biotec, Auburn, CA) and LS columns (cat#130-042-401, Miltenyi Biotec). CAR^+^ T cells were cryopreserved at days 14, 21, 28, and 35 (where applicable). Phenotyping and functional analyses were performed between days 21 to 29. ROR1-specific CAR^+^ T cells from patients with CLL diagnosis were derived in the same manner, except that tumor cells were depleted the day following electroporation with CD19 microbeads (cat# 130–019–301; Miltenyi) and then stimulated at above. Mock electroporated “No DNA” T cells were stimulated with OKT3-loaded AaPC clone#4 from each donor/patient for negative controls as previously described [36].

### Abundance and identity of mRNA molecules by digital profiling

At designated times after co-culture on AaPC and cytokines, T cells were lysed with 160 μL RLT Buffer (Qiagen, Valencia, CA) per 10^6^ cells and lysates were frozen at -80°C. RNA lysates were thawed and immediately analyzed using nCounter Analysis System (NanoString Technologies, Seattle, WA) with “lymphocyte codeset array” (LCA). LCA data was normalized to both spike positive control RNA and housekeeping genes (*ACTB*, *G6PD*, *OAZ1*, *POLR1B*, *POLR2A*, *RPL27*, *RPS13*, and *TBP*) as detailed previously [37] and normalized counts were reported. Limit-of-detection (LOD) was calculated from the negative control counts and reported as the mean plus two-times the standard deviation (mean+2xSD) and shown as dashed lines in graphs of mRNA data.

### Flow cytometry and Immunohistochemistry

All mAbs were purchased from BD Biosciences (San Jose, CA), except for CCR7 mAb (eBioscience, San Diego, CA), IL-15 mAb (R&D Systems, Minneapolis, MN), Fc mAb used to detect CAR (Invitrogen), and 4A5 mAb used to detect ROR1 (Kipps TJ laboratory, UCSD). Staining was performed as described [38]. Samples for flow cytometry were acquired on FACS Calibur (BD Biosciences) and analyzed with FlowJo software (version 7.6.3). For immunohistochemistry, fresh frozen sections of malignant and normal pancreas were stained with 100 μg/ml of 4A5 mouse-anti-human ROR1 mAb or control mouse IgG2b mAb (isotype), and developed using an anti-mouse secondary antibody conjugated with horse radish peroxidase. Tissues were visualized with diaminobenzidine (DAB) and counterstained with hematoxylin. Slides were evaluated by the study pathologist to identify the tissue or cell-type stained and intensity of staining.

### *In vitro* functional assays

*In vitro* specific lysis was assessed using a standard 4-hour chromium release assay, as previously described [32]. Expression of cytokines was evaluated by intracellular staining and flow cytometry. CAR^+^ T cells were incubated with an equal volume and number of target cells for 6 hours at 37°C in the presence of Brefeldin-A (GolgiPlug; BD Biosciences) to block exocytosis and secretion of cytokines. Co-cultures were then (i) stained for surface markers, *e*.*g*., CD3 and CAR (Fc-specific antibody), (ii) fixed and permeabilized with BD Cytofix/Cytoperm (cat# 555028, BD Biosciences), (iii) stained for intracellular IFNγ, and (iv) analyzed by flow cytometry.

### Mouse experiments

Kasumi-2-*ffLuc*-mKate tumor cells were generated according to a protocol described elsewhere [37]. Transduced Kasumi-2 cells were sorted for uniform mKate expression by FACS to obtain cells with *ffLuc* activity suitable for non-invasive BLI. Immunocompromised female NSG mice (6–12 weeks of age; NOD.Cg-Prkdc^scid^Il2r^gtm1Wjl^/SzJ; Jackson Laboratory, Bar Harbor, ME) were intravenously (i.v.) injected with 4x10^4^ Kasumi-2-ffLuc-mKate cells (designated day 0), and the following day mice (total n = 17) were mixed together then randomly distributed into 3 groups, which were treated with (i) no treatment (n = 5), (ii) ROR1RCD28^+^ T cells (n = 5), and (iii) ROR1RCD137^+^ T cells (n = 5). Mice injected with only T cells served as controls for xenogeneic graft-versus-host-disease (one mouse per T-cell group). T-cell doses (10^7^ total cells per mouse) were administered on days 1, 8, and 15. CAR expression on infused T cells was 96%, 91%, and 90% for ROR1RCD28 and 94%, 62%, and 46% for ROR1RCD137 on days 1, 8, and 15, respectively. IL-2 (60,000 IU) was intraperitoneally administered the day of T-cell dosing and the following day. BLI from tumor *ffLuc* was monitored twice per week. Log-rank (Mantel-Cox) test was used for statistical analysis between groups of mice (n = 5 per group).

## Results

### ROR1 is expressed on subsets of tumor cells, but not healthy pancreas

**Fig 1:**
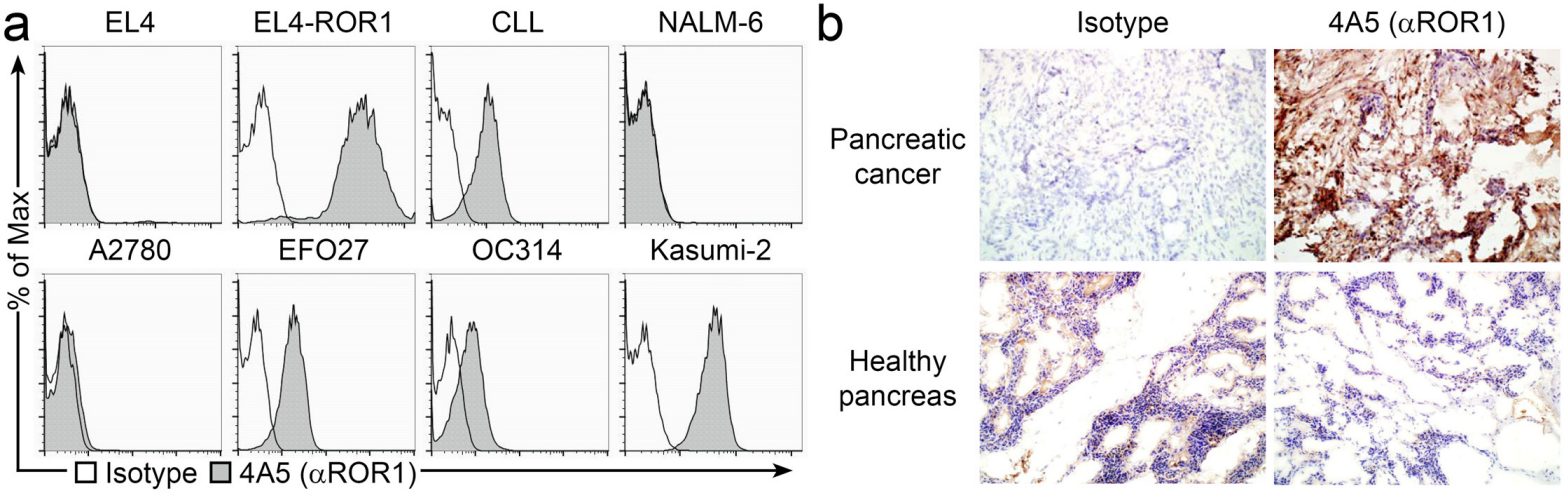
Evaluation of ROR1 expression on tumor cells, pancreatic cancer, and healthy pancreas. (a) Flow cytometry plots of samples stained with 4A5 mouse-anti-human ROR1 mAb (filled histograms) or matched isotype control (open histograms). Cell types tested were: EL4 murine T-cell lymphoma cell line, EL4 cells genetically modified to express ROR1 (EL4-ROR1), PBMC from patient with CLL diagnosis, non-t(1;19) B-ALL cell line NALM-6, t(1;19) B-ALL cell line Kasumi-2, and ovarian cancer cell lines A2780, EFO27, and OC314. (b) Fresh frozen sections of malignant and normal pancreas were stained with 4A5 mAb or isotype mAb, and developed using an anti-mouse secondary antibody conjugated with horse radish peroxidase. Tissues were visualized with diaminobenzidine and counterstained with hematoxylin. Slides were evaluated by the study pathologist to identify the tissue or cell-type stained and intensity of staining.

Before constructing CARs from 4A5, a mAb with specificity for human ROR1 [18–20, 24], we sought to validate its staining of tumor cells. The murine T-cell lymphoma cell line EL4 did not express ROR1 but following genetic modification with a ROR1 transposon this cell line showed bright staining for ROR1 (Fig 1a). Consistent with other reports, all of the CLL samples that we tested were ROR1^+^ as measured by 4A5 mAb staining (representative example is displayed). The t(1;19) B-ALL cell line Kasumi-2 expressed ROR1 whereas the non-t(1;19) B-ALL cell line NALM-6 did not. Eleven of twelve ovarian cancer cell lines tested positive for ROR1 (EFO27 and OC314 are shown as examples) and A2780 was the only ovarian cancer cell line we evaluated that did not express ROR1. Previous reports have identified ROR1 mRNA in the pancreas [39], so we tested 4A5 mAb staining against healthy and malignant pancreatic tissue using immunohistochemistry (Fig 1b). We were unable to detect ROR1 expression in healthy pancreas but ROR1 was widely and strongly expressed by pancreatic cancer tissue. Thus, this staining dataset corroborated our rationale to use the 4A5 mAb to construct CARs specific for ROR1 expressed on tumor cells.

### *Sleeping Beauty* transposition and co-culture on ROR1^+^ AaPC with cytokines generates ROR1-specific T cells

**Fig 2:**
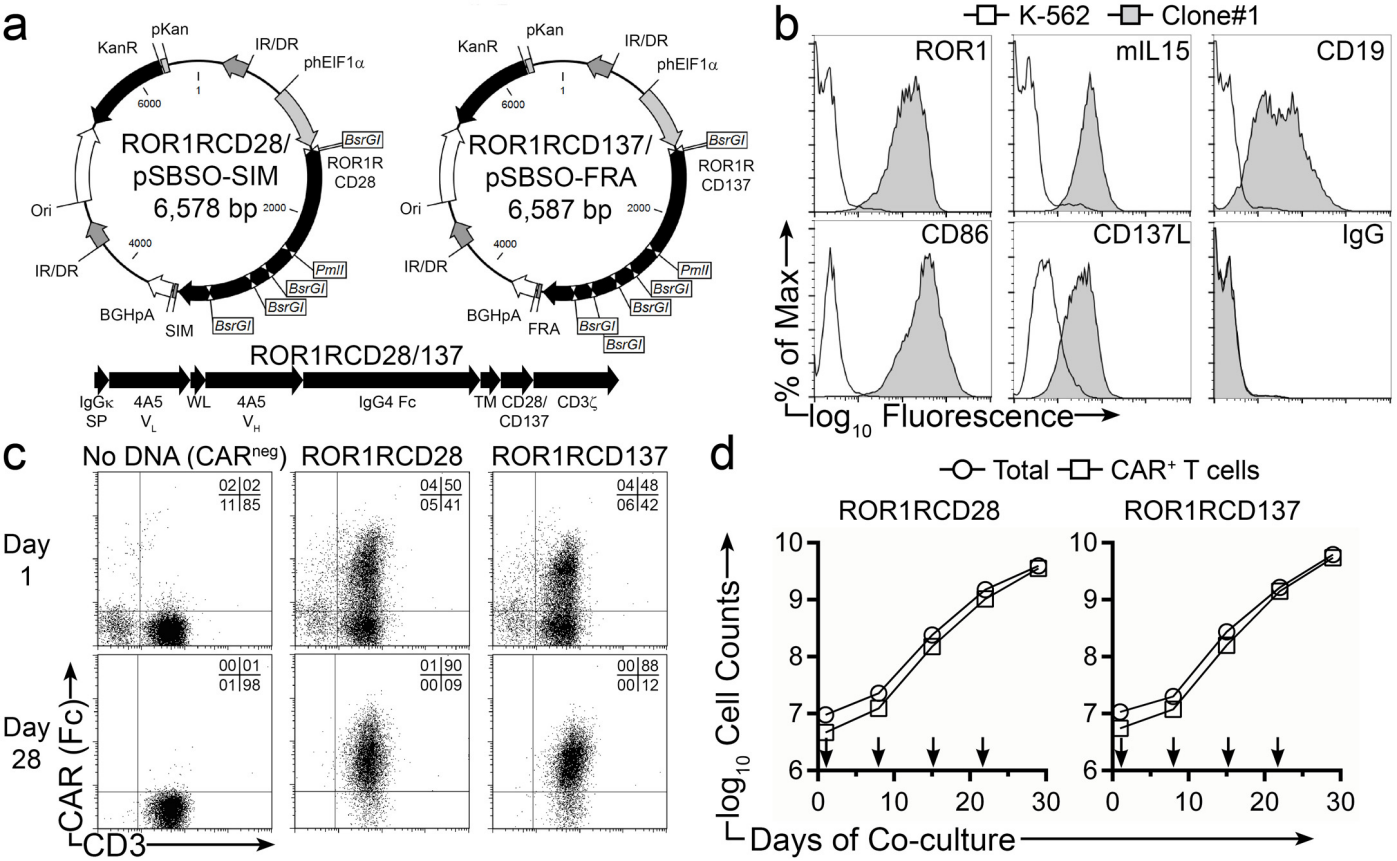
Sustained numeric expansion of ROR1-specific CAR^+^ T cells upon clone#1 AaPC and cytokines. (a) DNA plasmid vector maps for ROR1RCD28/pSBSO-SIM and ROR1RCD137/pSBSO-FRA. Abbreviations are IR/DR: *Sleeping Beauty* Inverted Repeat/Direct Repeat, phElF1α: Human Elongation Factor-1-α region hybrid promoter, ROR1RCD28: Human codon-optimized ROR1-specific scFv:Fc:CD28:CD3ζ CAR, ROR1RCD137: Human codon-optimized ROR1-specific scFv:Fc:CD137:CD3ζ CAR, SIM: “SIM” PCR tracking oligonucleotides, FRA: “FRA” PCR tracking oligonucleotides, BGHpA; bovine growth hormone polyadenylation sequence, Ori: minimal *E*.*coli* origin of replication, KanR: Bacterial selection gene encoding Kanamycin resistance, pKan: prokaryotic Kanamycin promoter. Digestion with *BsrGI* enzyme can distinguish the two plasmids, which have high degrees of similarity, and with *PmlI* enzyme can distinguish them from CD19RCD28 plasmid, which does not have *PmlI* site. The entire plasmid sequences were verified by Sanger-based sequencing techniques. (b) Parental K-562 (open histograms) and clone#1 AaPC (shaded histograms) were stained for CD19, CD86, CD137L, ROR1, and IL15 (membrane-bound IL-15; mIL15) where isotype (IgG) was used a negative control. (c) Expression CAR in ROR1RCD28 (middle) and ROR1RCD137 (right) T cells the day following electroporation (top panels) and after 28 days of co-culture on clone#1 AaPC (bottom panels) where “no DNA” T cells (left) were used as negative controls. T cells were marked by CD3 staining and CAR^+^ cells were detected with Fc-specific antibody. Quadrant frequencies are displayed in upper right corners. (d) Proliferation kinetics of total cells (circles) and CAR^+^ T cells (squares) on clone#1 AaPC over 28 days of co-culture. ROR1RCD28 displayed on the left and ROR1RCD137 shown on the right. Arrows represent addition of γ-irradiated clone#1 AaPC. Data are representative of 3 donors expanded in 3 independent experiments. Expansion data can be found in S1 Dataset, Fig 2 tab.

SB transposition followed by co-culture with AaPC, IL-2, and IL-21 was employed to propagate T cells expressing a 2^nd^ generation CD19-specific CAR that activates via CD3ζ/CD28 endodomain (designated CD19RCD28) and these T cells have been infused into patients with B-cell malignancies [28–33, 40]. This strategy was adapted to target ROR1 by replacing the CD19-specific scFv sequence in the CAR with the scFv sequence derived from 4A5 mAb. Two SB transposons were constructed for side-by-side comparison of 2^nd^ generation ROR1-specific CARs that differ in signaling via CD28 (ROR1RCD28) or CD137 (ROR1RCD137) (Fig 2a). The remaining portions of the CARs, *e*.*g*., murine IgGκ signal peptide, Whitlow linker, modified human IgG_4_-Fc stalk, human CD28 transmembrane, and human CD3ζ intracellular domains (with three ITAM activation signals) were identical between the two constructs. We used AaPC (designated clone#1) to achieve the selective expansion of CAR^+^ T cells. These feeder cells were derived from K-562 cells genetically modified to co-express ROR1, CD19, CD86, CD137L, and a membrane-bound IL-15/IL-15-Receptor-α (IL15Rα) fusion protein (mIL15) (Fig 2b). Peripheral blood mononuclear cells (PBMC) from healthy donors were co-electroporated with DNA plasmids expressing SB11 transposase and either ROR1RCD28 or ROR1RCD137 transposons. The following day, expression of CARs (from both integrated and episomal transgenes) was detected in ROR1RCD28^+^ and ROR1RCD137^+^ T cells at 41% ± 6% and 41% ± 8% (mean ± SD; n = 3), respectively, as evidenced by co-staining for Fc (the IgG_4_-Fc extracellular stalk of CAR) and CD3 (Fig 2c top). Sham-electroporated “No DNA” T cells were co-cultured with γ-irradiated OKT3-loaded clone#4 AaPC, IL-2, and IL-2 and served as negative controls [36]. Co-cultures of CAR^+^ T cells and γ-irradiated clone#1 AaPC were then initiated in the presence of exogenous IL-2 and IL-21 in parallel to the “No DNA” co-cultures. Recursive stimulations of AaPC, IL-2, and IL-21 were performed every 7 days for a total of four stimulations, and IL-2 and IL-21 were replaced every 2–3 days during co-culture. At day 28 of co-culture, CAR was expressed in T cells at 90% ± 3% and 79% ± 11% (mean ± SD; n = 3) for ROR1RCD28 and ROR1RCD137, respectively (Fig 2c bottom). These two CAR-expressing T cell populations appear to proliferate at similar rates, as noted by the total numbers of cells counted at the end of culture (p = 0.66; Two-way ANOVA), and in number of CAR^+^ T cells generated (p = 0.74). Total cell numbers closely coincided with CAR^+^ T-cell counts for both ROR1RCD28 and ROR1RCD137, resulting in production of at least 10^9^ CAR^+^ T cells (Fig 2d). In similar studies, CLL patient-derived PBMC, composed of >91% CD19^+^ malignant cells, were electroporated with SB11 and ROR1-specific CAR SB transposons. The following day, cultures were depleted of malignant cells with CD19 paramagnetic microbeads and stimulated weekly with clone#1 AaPC in the presence of IL-2 and IL-21, as was performed for healthy donor PBMC. This resulted in >160-fold expansion of CAR^+^ T cells with 92% and 80% expressing ROR1RCD28 and ROR1RCD137, respectively. This indicates that autologous ROR1-specific T cells can be generated from recipients with advanced CLL. Thus, SB transposition and co-culture on AaPC clone#1 with IL-2/-21 generated clinically-appealing quantities of ROR1-specific T cells with high frequencies of CAR expression.

### Electroporated and propagated CAR^+^ T cells have a transcriptional profile consistent with diverse memory phenotypes and potential for effector functions

**Fig 3:**
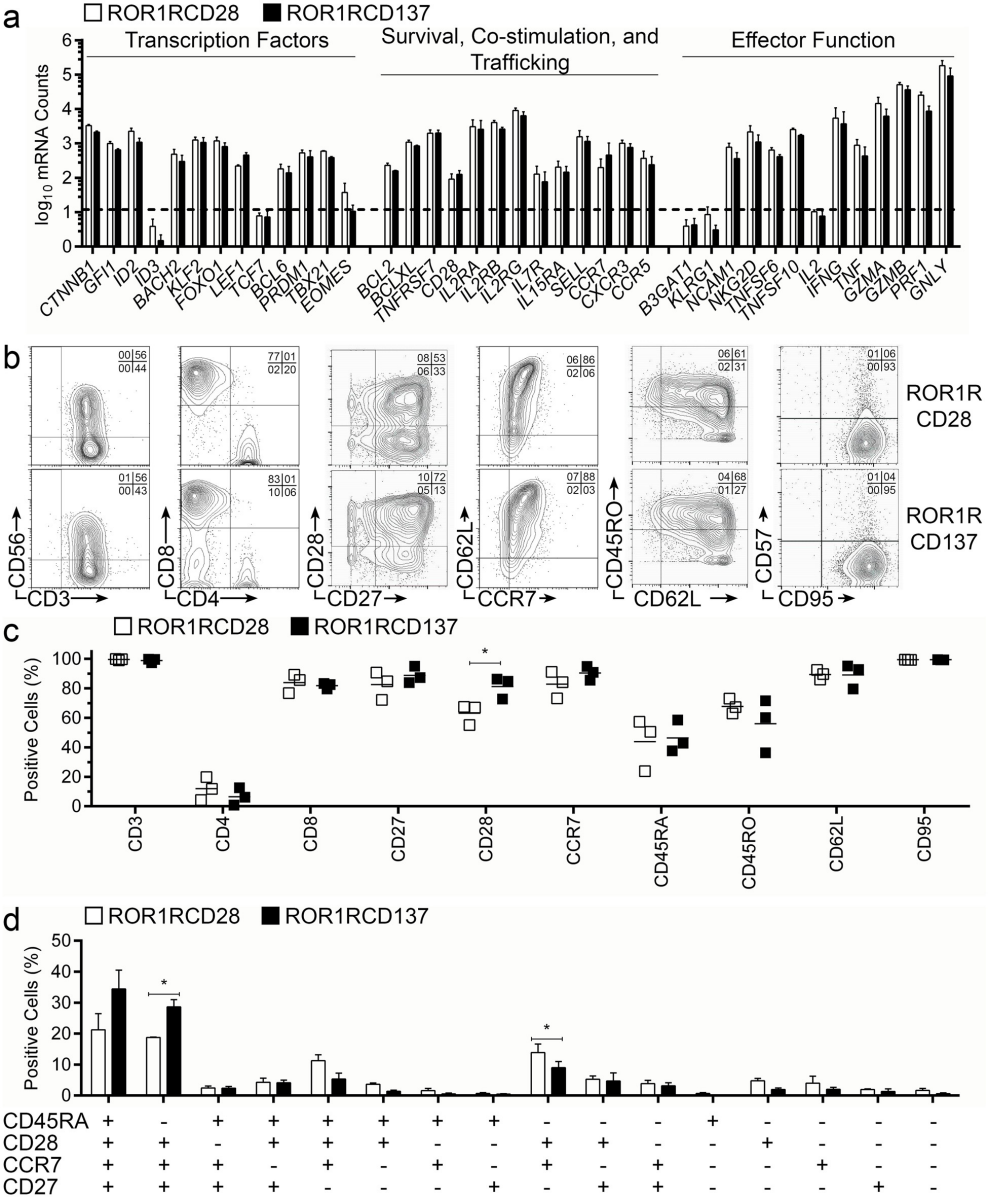
Lymphocyte transcriptional profile and memory markers expressed on CAR^+^ T-cell surface. After 29 days of expansion on clone#1 AaPC/IL-2/IL-21, ROR1RCD28 and ROR1RCD137 cells were (i) lysed for mRNA expression analysis using bar-codes or (ii) phenotyped for T-cell surface markers by flow cytometry. (a) RNA lysates were profiled for expression of a selected group of lymphocyte genes with non-enzymatic digital multiplex array of mRNA transcripts (NanoString) where transcription factors are shown on the left, genes associated with survival, co-stimulation, and trafficking are shown on the middle, and genes associated with effector function are shown on the right. Dashed line represents the limit-of-detection calculated by mean + 2xSD of negative controls. (b) Flow cytometry of ROR1RCD28 and ROR1RCD137 T cells showing co-staining for CD3 and CD56, CD4 and CD8, CD28 and CD27, CD62L and CCR7, CD45RO and CD62L, or CD95 and CD57 in cells gated for CAR expression based staining with Fc-specific antibody. ROR1RCD28^+^ T cells are shown in the top panels and ROR1RCD137 T cells are displayed in the bottom panels. One of 3 representative donors is displayed and quadrant frequencies are shown in the upper right corners. (c) Cumulative frequencies of cells staining positive for each memory marker within an extended memory phenotype panel. (d) Multi-parameter flow cytometry was used to determine frequencies of cells staining positive for combinations of CD45RA, CD27, CD28, and CCR7. For (c) and (d) ROR1RCD28 are in open shapes/bars and ROR1RCD137 are in closed shapes/bars and lines displayed in (c) are means (n = 3) and in (d) are mean ± SD (n = 3). Student’s two-tailed t-tests were used for statistical analysis between the two groups. *p<0.05 Expression and phenotype data can be found in S1 Dataset, Fig 3 tab.

T-cell memory phenotype can be used to predict proliferation, survival, and effector potential of an infused CAR^+^ T-cell product. We used non-enzymatic digital multiplex array of mRNA transcripts (NanoString) to assess the transcriptional profile of CAR^+^ T cells following numeric expansion with AaPC and cytokines. Transcription factors associated with less differentiated T-cell phenotype, *e*.*g*., *CTNNB1* (β-Catenin), *GFI1* (Growth Factor Independence-1), *ID2* (Inhibitor of DNA Binding-2), *BACH2* (BTB and CNC Homology-2), *KLF2* (Kruppel-like Factor-2), *FOXO1* (Forkhead Box-O1), and *LEF1* (Lymphoid Enhancer Binding Factor-1), were expressed by populations of ROR1-specific T cells concurrently with transcription factors associated with T cells in later memory stages, including *BCL6* (B-cell Lymohoma-6), *PRDM1* (BLIMP-1), and *TBX21* (T-bet), suggesting that the outgrowth of CAR^+^ T cells was heterogeneous in memory gene regulation (Fig 3a left). Quantities of transcription factors *ID3* (Inhibitor of DNA Binding-3; reciprocal and upstream to *ID2*), *TCF7* (T-cell Factor-1; TCF1; reciprocal to and upstream of *LEF1*), and *EOMES* (Eomesodermin; similar in function to T-bet) were at or below the limit-of-detection, indicating that coordination through some of the gene regulation cascades had occurred during or prior to expansion on AaPC. Thus, CAR^+^ T cells had a continuum of memory transcription factor expression. Genes associated with intrinsic memory T-cell survival, *e*.*g*., *BCL2* (B-cell Lymphoma-2) and *BCLXL* (BCL2-related protein long isoform), and receptors for cytokines responsible for extrinsic memory T-cell survival and proliferation, *e*.*g*., *IL2RA* (IL-2-Receptor-α; CD25), *IL2RB* (IL-2-Receptor-β; CD122), *IL2RG* (IL-2-Receptor-γ; CD132), *IL7R* (IL-7-Receptor-α; CD127), and *IL15RA* (IL-15-Receptor-α), were detected in CAR^+^ T cells suggesting that persistence and survival of these cells following adoptive transfer is plausible (Fig 3a middle). Memory-associated T-cell trafficking molecules, *e*.*g*., *SELL* (L-Selectin; CD62L), *CCR7* (CCR7), *CXCR3* (CXCR3), and *CCR5* (CCR5), and lymphocyte co-stimulatory molecules, *e*.*g*., *TNFRSF7* (CD27) and *CD28* (CD28), were detected in ROR1RCD28^+^ and ROR1RCD137^+^ T cells, indicating that the CAR^+^ T cells may have been derived from or developed into T-cell memory cells. Importantly, genes associated with exhaustion and terminal differentiation, including *B3GAT1* (Beta-1,3-Glucuronyltransferase-1; CD57) and *KLRG1* (KLRG1) were not expressed above the limit-of-detection by the CAR^+^ T cells (Fig 3a right). Genes encoding surface receptors leading to killer function, *e*.*g*., *NCAM1* (Neural cell adhesion molecule-1; CD56) and *NKG2D* (NKG2D), cytokines with pro-inflammatory properties, *e*.*g*., *IFNG* (Interferon-γ) and *TNF* (Tumor Necrosis Factor), and molecules responsible for directing cell death and/or cytolysis, *e*.*g*., *TNFSF6* (Fas-ligand), *TNFSF10* (TRAIL), *GZMA* (Granzyme-A), *GZMB* (Granzyme-B), *PRF1* (Perforin-1), and *GNLY* (Granulysin), were expressed by CAR^+^ T cells suggesting that these cells could have effector function in addition to their potential for memory formation. In aggregate, CAR^+^ T cells which emerge after recurrent additions of AaPC and IL-2/IL-21 appear to contain sub-populations with desirable gene expression predictive of therapeutic efficacy after adoptive transfer.

### CAR^+^ T cells express surface markers consistent with T-cell memory

Flow cytometry was used to validate the NanoString panel, examine the surface expression of canonical T-cell markers, and determine the frequencies of memory populations with multi-parameter staining. CAR^+^ T-cell populations expressed CD56 (*NCAM1*), although CD3^neg^CD56^+^ NK cells were absent from final cultures (Fig 3b, first panels). A preponderance of CD8^+^ T cells was present in the cultures relative to CD4^+^ T cells (Fig 3b, second panels), and >99% of cells expressed CD3. High frequencies of CAR^+^ T cells were found co-expressing CD28 and CD27 co-stimulatory molecules (Fig 3b, third panels), which are characteristics of T-cell memory. CD27, in particular, has been correlated to durable regressions resulting from CD8^+^ T-cell treatments [41]. The stemness of CD8^+^ memory cells has been linked to expression of L-selectin [12], which was detected in CAR^+^ T cell’s mRNA (*SELL*) and as protein (CD62L), and was concurrently expressed with the associated lymphoid homing marker CCR7 (Fig 3b, fourth panels). Thus, such CAR^+^ T cells with long-lived potential could migrate to secondary lymphoid structures harboring ROR1^+^ leukemias. Few CD45RO^+^CD62L^neg^ cells were detected, which are generally associated with T_EM_ or late-stage effector memory cells, and could be contrasted to the larger abundance of CD45RO^neg^CD62L^+^ cells usually associated with less differentiated T cells (Fig 3b, fifth panels). Virtually all cells expressed CD95, a marker for activation and differentiation beyond T_N_ lineages, which was expected on T cells cultured *ex vivo*, but the same T cells did not express CD57, a marker of exhaustion (Fig 3b, sixth panels). In aggregate, the staining of these markers was consistent amongst donors (Fig 3c). Multi-parameter flow cytometry revealed that most electroporated/propagated T cells belonged to less-differentiated memory phenotype primarily composed of T_SCM_ (CD45RA^+^CD27^+^CD28^+^CCR7^+^) and T_CM_ (CD45RA^neg^CD27^+^CD28^+^CCR7^+^) (Fig 3d) [42, 43]. There were appreciable frequencies of CD45RA^+^CD27^neg^CD28^+^CCR7^+^ and CD45RA^neg^CD27^neg^CD28^+^CCR7^+^, which we were not able to define as a specific memory subset, and almost no T_EMRA_ cells (CD45RA^+^CD27^neg^CD28^neg^CCR7^neg^). In aggregate, the surface phenotype of ROR1-specific CAR^+^ T cells corroborated the mRNA digital barcoding profiling data (NanoString) and indicated that these cells have desirable characteristics for fighting ROR1^+^ malignancies.

### ROR1-specific CAR^+^ T cells produce IFNγ in response to ROR1^+^ tumor cells

**Fig 4:**
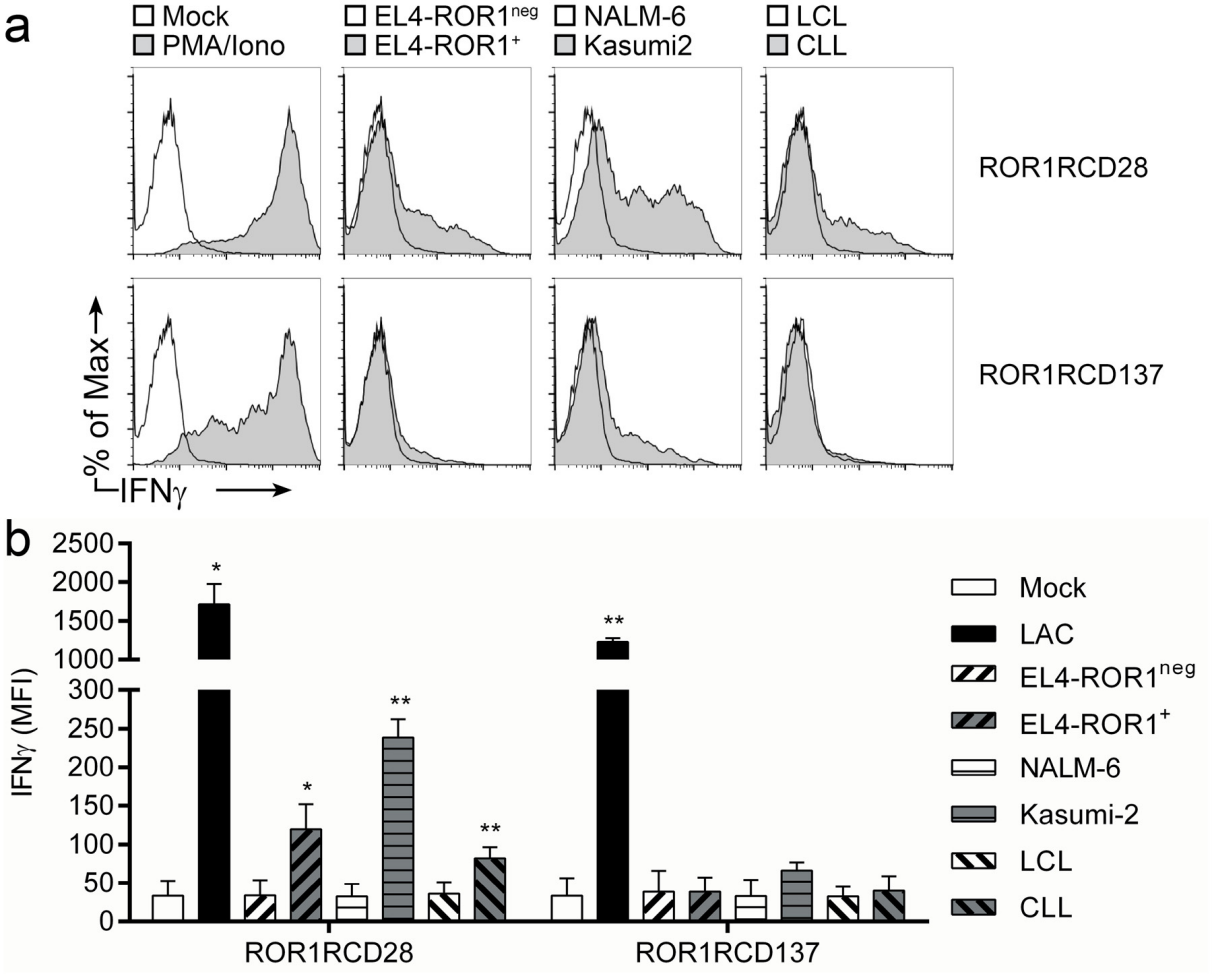
IFNγ production by ROR1-specific CAR^+^ T cells in response to ROR1^+^ targets. At day 29 of co-culture with AaPC/IL2/IL-21, CAR^+^ T cells were co-cultured for 6 hours at 37°C with tumor targets or non-specific mitogenic stimuli (PMA/Iono) then analyzed for expression of IFNγ in CAR^+^ T cells (gated based on Fc expression). Brefeldin-A (GolgiPlug) was added to T cells to block IFNγ secretion. (a) Representative flow cytometry plots where ROR1RCD28 cultures are on the top and ROR1RCD137 cultures are on the bottom. Percentage of max (y-axes) normalized total cell numbers in each sample for consistency between samples so that IFNγ fluorescent intensity (x-axes) could be compared between conditions. (b) Cumulative mean fluorescence intensities (MFI) of IFNγ staining from co-cultures where mean ± SD (n = 3 donors) is shown. Student’s paired, 1-tailed t-test for statistical analysis between target co-culture and T cells alone (Mock; media only). *p<0.05 and **p<0.01 IFNγ data can be found in S1 Dataset, Fig 4 tab.

We monitored for the production of IFNγ by intracellular cytokine staining of healthy donor CAR^+^ T cells following 6 hour co-culture with target cells. Both ROR1RCD28^+^ and ROR1RCD137^+^ T cells produced significant quantities of IFNγ when treated with PMA/Ionomycin (non-specific mitogenic stimulus; positive control) relative to mock-activated (media only; negative control) cultures (Fig 4). ROR1RCD28^+^ T cells stained brightly for IFNγ when co-cultured with ROR1^+^ tumor targets EL4-ROR1, Kasumi-2, and CLL cells, but did not produce IFNγ, as evidenced by similar staining of unchallenged cultures (mock; media only) to co-cultures with ROR1^neg^ cells from EBV-transformed healthy donor B-cell lymphoblastoid cell lines (LCL), NALM-6, and parental EL4 cells (Fig 4a top). IFNγ production by ROR1RCD137^+^ T cells slightly increased only in response to Kasumi-2 cells, demonstrating that there were differences in the effector cytokine production of the two CAR populations to certain tumor cells (Fig 4a bottom). In summary, effector function of CAR^+^ T cells, as manifested by IFNγ expression, was restricted to ROR1^+^ tumor cells and ROR1RCD28^+^ T cells produced more IFNγ than did ROR1RCD137^+^ T cells in response to ROR1.

### ROR1-specific killing by CAR^+^ T cells *in vitro*

**Fig 5:**
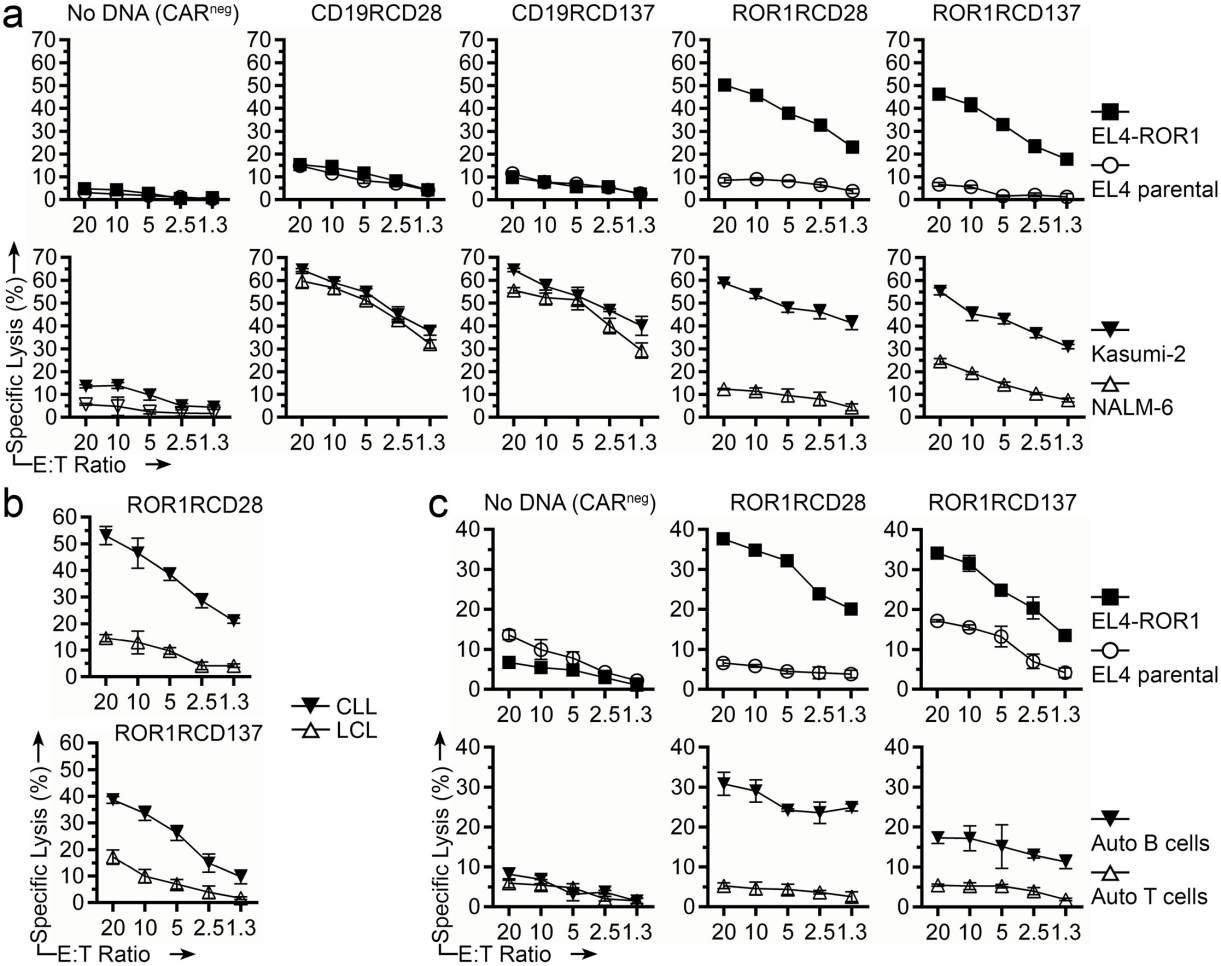
Specific cytolysis of ROR1^+^ tumor cells by CAR^+^ T cells. Four-hour chromium release assay was used to assess specific lysis by T cells at decreasing effector to target (E:T) ratios. (a) No DNA (CAR^neg^), CD19RCD28^+^, CD19RCD137^+^, ROR1RCD28^+^, or ROR1RCD137^+^ T cells generated from healthy donor PBMC were challenged with EL4-ROR1^+^ cell line (closed squares), EL4 parental (ROR1^neg^) cell line (open circles), Kasumi-2 cell line (closed inverted triangles), or NALM-6 cell line (open triangles). (b) CAR^+^ T cells generated from healthy donor PBMC were challenged with allogeneic CLL cells (closed inverted triangles), healthy donor allogeneic B-cell LCL (open triangles). (c) No DNA (CAR^neg^), ROR1RCD28^+^, or ROR1RCD137^+^ T cells generated from CLL patient PBMC were challenged with EL4-ROR1^+^ cell line (closed squares), EL4 parental (ROR1^neg^) cell line (open circles), autologous B cells (closed inverted triangles), or autologous T cells (open triangles). Data are mean ± SD of triplicate measurements in CRA and are representative of four donors from four independent experiments. Lysis data can be found in S1 Dataset, Fig 5 tab.

The killing of tumor cells by genetically modified T cells is another measurement of re-directed specificity. Four-hour chromium release assay was used to assess the specific lysis of tumor-cell lines and primary tumor cells. ROR1RCD28^+^ and ROR1RCD137^+^ T cells derived from healthy donor PBMC efficiently lysed EL4-ROR1^+^ and Kasumi-2 but showed minimal lysis of EL4 parental (ROR1^neg^) and NALM-6 cells (Fig 5a). Furthermore, autologous CD19RCD28^+^ and CD19RCD137^+^ T cells displayed minimal lysis of EL4 and EL4-ROR1 but lysed both Kasumi-2 and NALM-6 (both CD19^+^) suggesting that the ROR1RCD28^+^ and ROR1RCD137^+^ T cells were more discriminant in their targeting of these B-ALL cell lines (Fig 5a bottom). Autologous CAR^neg^ T cells (No DNA) showed no lysis of these 4 cell lines indicating that the CAR responses were specific. ROR1RCD28^+^ and ROR1RCD137^+^ T cells generated from healthy donor PBMC also lysed allogeneic CLL patient B cells (ROR1^+^), but spared allogeneic LCL (ROR1^neg^) derived from healthy donor B-cells (Fig 5b). Similarly, ROR1RCD28^+^ and ROR1RCD137^+^ T cells generated from PBMC of a patient with CLL were able to lyse EL4-ROR1 and autologous tumor cells (ROR1^+^; isolated with CD19-specific magnetic beads), while autologous T cells and parental EL4 (both ROR1^neg^) cells were spared (Fig 5c). In summary, ROR1-specific CAR^+^ T cells demonstrated effective *in vitro* specific lysis of ROR1^+^ tumor cells in both autologous and allogeneic settings.

### *In vivo* clearance of leukemia by ROR1-specific CAR^+^ T cells

**Fig 6:**
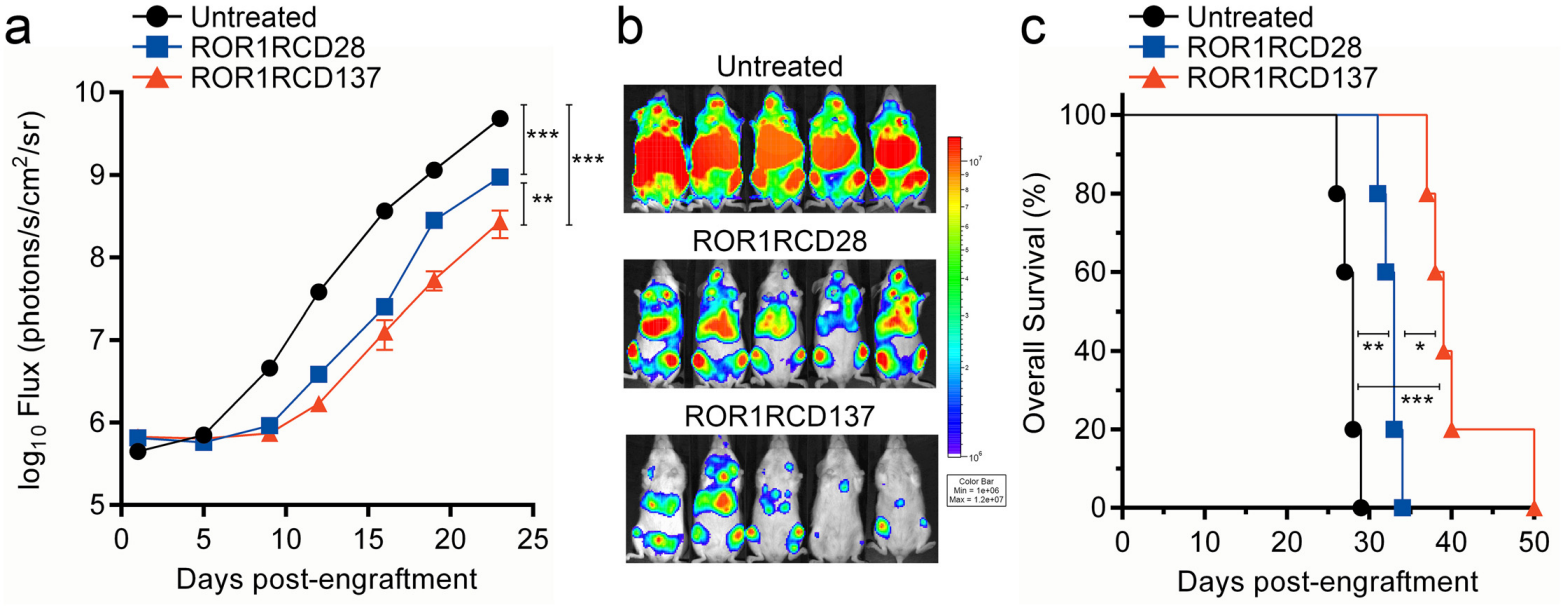
*In vivo* tumor clearance by ROR1-specific CAR^+^ T cells. NSG mice were intravenously (i.v.) injected with Kasumi-2-*ffLuc*-mKate cells and were treated with three i.v. doses of T cells to assess the ability of ROR1-specific T cells to manage disease. High-dose IL-2 was added intraperitoneally the day of injection and the following day. (a) Non-invasive BLI reported as flux was serially measured in untreated (black circles), ROR1RCD28^+^ T cell-treated (blue squares), and ROR1RCD137^+^ T cell-treated (red triangles) mice. Data are mean ± SEM (n = 5). Two-way ANOVA was used for statistical analysis. (b) Representative BLI images at day +23 post-tumor cell engraftment. (c) Overall survival of mice. *p<0.05, **p<0.01, ***p<0.001 BLI and survival data can be found in Supporting Information, Fig 6 tab.

In order to evaluate the anti-tumor activity of ROR1-specific CAR^+^ T cells *in vivo*, a xenograft model of leukemia was established in immunocompromised mice and treated with ROR1-specific CAR^+^ T cells. As Kasumi-2 cells were sensitive to ROR1-specific T-cell lysis and were not lysed by autologous CAR^neg^ T cells (Fig 5), they were genetically modified with lentivirus particles to introduce mKate red fluorescence protein for sorting transduced cells and enhanced *firefly luciferase* (*ffLuc*) [44] for serial non-invasive bioluminescence imaging (BLI) of tumor burden *in vivo*. NOD.*scid*.γ_c_^-/-^ (NSG) mice were used because they lack functional adaptive immune systems and can therefore accept human tumor xenografts. Healthy, 6–12 week old female NSG mice were engrafted with Kasumi-2-*ffLuc*-mKate and distributed into 3 treatment groups (n = 5 per group): (i) no treatment, (ii) ROR1RCD28^+^ T-cell treated, and (iii) ROR1RCD137^+^ T-cell treated, where T cells were given at days 1, 8, and 15 post-tumor cell engraftment (Fig 6). Untreated mice had consistent log_10_-fold increases in bioluminescence flux from their tumors (Fig 6a black circles and 6b top) and succumbed to disease after an average of 27 days following engraftment (Fig 6c black circles). ROR1RCD28^+^ T cells were able to diminish tumor burden compared to untreated mice as measured by tumor BLI flux (Fig 6a blue squares and 6b middle) and prolonged survival compared to untreated mice (Fig 6c blue squares). ROR1RCD137^+^ T cells eliminated tumor burden above both untreated mice and ROR1RCD28^+^ T cell-treated mice (Fig 6c red triangles and 6b bottom), and were able to increase survival compared to both untreated mice and ROR1RCD28^+^ T cell-treated (Fig 6c red triangles). In summary, ROR1-specific CAR^+^ T cells can efficiently treat ROR1^+^ leukemia in mice which supports testing of these T cells in the clinic as an investigational therapy for patients with ROR1^+^ malignancies.

## Discussion

This work reveals the pre-clinical basis to support our current “first-in-human” Phase I clinical trial of CAR^+^ T cells for investigational therapy of ROR1^+^ malignancies. We demonstrate that SB transposition achieved stable CAR expression in T cells and, in concert with co-culture on clone#1 AaPC, resulted in heterogeneous outgrowth of T-cell memory populations with ROR1-restricted anti-tumor activity. Currently, this process takes 28 days to achieve clinical numbers of T cells. Potentially shortening the production time for cell growth may (i) simplify the overall process, (ii) preserve more of the minimally differentiated subsets, and (iii) could take advantage of *in vivo* proliferation of CAR^+^ T cells thereby augmenting therapeutic efficacy. As SB transposition was used to express ROR1-specific CARs in naïve or minimally-differentiated T-cell populations present in quiescent PBMC, as has been done with other SB-based CAR studies [31, 38], the prospect of giving T cells earlier in the proliferative cycle is of great interest to our group. Homeostatic cytokines, *e*.*g*., IL-15, can promote the survival of less differentiated T cells, and trans-presentation of IL-15 by IL15Rα has higher signaling potency than IL-15 alone [45, 46]. Thus, we chose to introduce mIL15 (IL-15/IL-15Rα fusion protein) rather than IL-15 tethered to a hinge/Fc stalk, which is the moiety expressed on AaPC used to generate CAR^+^ T cells expressing CD19RCD28 [47], on the clone#1 AaPC. The AaPC feeder platform, which is amenable to genetic introduction of TAAs (*e*.*g*., ROR1) and molecules that promote T-cell survival (*e*.*g*., mIL15), in concert with SB transposition can support the propagation of populations of T cells predicted to have sustained engraftment, *i*.*e*., T_SCM_ and CD62L^+^ T_CM_ populations [12, 48], while enforcing CAR expression on T cells through recursive antigen exposure; therefore, they are attractive platforms for the generation of ROR1-specific T cells for cancer immunotherapy.

The choice of signaling motif within a 2^nd^ generation CAR endodomain may impact the therapeutic activity of infused T cells. CD19-specific CARs activating T cells via chimeric CD28 or CD137 endodomains led to objective clinical responses [3–7]. We designed CARs that used the same extracellular structure in order to reveal differences arising between the endodomains. The most notable differences were that ROR1RCD137^+^ T cells displayed enhanced *in vivo* tumor clearance (Fig 6) despite showing reduced IFNγ production *in vitro* relative to ROR1RCD28^+^ T cells (Fig 4). The decrease in IFNγ-response with CARs signaling through CD137 relative to those signaling through CD28 is consistent with independent studies comparing ROR1-specific CARs [25, 39, 49, 50]. These studies introduced CARs derived from different ROR1-specific antibodies (2A2 and R12) into T_CM_ cells with lentivirus and showed that CD137 signaling resulted in reduced cytokine production and increased anti-tumor activity *in vivo* [50]. CARs with short extracellular spacers, especially those without Fc stalks that could be bound by cells expressing CD64 (high-affinity Fc receptor), were optimal for other ROR1 CARs (2A2 and R12 moieties) [49]. Future studies from our group will evaluate whether truncation or replacement of the Fc stalk in CARs constructed from 4A5 mAb, introduced into T cells with SB, and cultured on AaPC will produce similar benefits. An inverse correlation between *in vitro* effector function (cytokine production and cytolysis) and *in vivo* tumor clearance by CD8^+^ tumor-specific T cells has also been established for adoptive immunotherapy of melanoma, further supporting the notion that *ex vivo* culture conditions and memory phenotype of infused T cells are useful parameters to correlate with therapeutic efficacy [11]. A side-by-side comparison of the two CARs using a competitive repopulation experiment in a clinical trial is likely needed to reveal which CAR design is superior for a given tumor and perhaps even a particular patient.

A major advantage of targeting ROR1 over the current T-cell therapies targeting CD19 is that recipients would not deplete B cells and develop hypogammaglobulinemia, thereby mitigating the risk for impaired humoral immunity [32, 51]. ROR1 was originally identified on the surface of CLL cells with absent expression on normal tissues, including hematopoietic cells [18, 24]. Subsequently, ROR1 was described on some acute leukemias and solid tumors [19, 20, 34]. The 4A5 ROR1-specific mAb (used in this study to construct the CAR) did not detect ROR1 in healthy tissues by either immunoblot or immunohistochemistry (Fig 1b) [18, 20]. The only healthy cells to be stained with 4A5 mAb are hematogones, which are rare pre-B-cells that do not appear to be obligate precursors to mature B cells [24]. There has been some discordance between the mRNA transcript data and protein staining data, which may be explained by off targeting of ROR2 gene, which is expressed after birth in many tissues, and is highly homologous to ROR1. Given that ROR1-specific T cells propagated in this study have potential for long-term engraftment due to their memory T-cell phenotype, it is plausible that patients treated with these genetically modified T cells could experience prolonged anti-tumor effects with potential for elimination of minimal disease or relapsed ROR1^+^ malignancies. As a control for adverse events, conditional suicide genes, *e*.*g*., inducible Caspase9, could be co-expressed with CAR in order to eliminate T cells *in vivo* as needed [52].

In summary, our data support the clinical application of ROR1-specific CAR^+^ T cells and a Phase I clinical trial is open to patients with CLL as of July 2014 (NCT02194374). Clinical-grade DNA plasmids coding for ROR1RCD137 and SB11 have been produced and a master-cell bank of AaPC clone#1 has been generated. Furthermore, regulatory documents and materials are in place to undertake the clinical trial. This will be the first time ROR1 on CLL has been targeted by T cells; therefore, the primary endpoint will be to determine toxicity and maximum tolerated T-cell dose. Future trials can then assess the clinical impact of targeting ROR1 on solid tumors.

## Supporting Information

S1 Dataset Raw data as Excel spreadsheet. (Fig 2 tab) Cell counts at designated times as measured by trypan blue exclusion. (Fig 3 tab) Normalized mRNA counts from NanoString array of T cells at day 29 of co-culture (top), surface phenotype of CAR^+^ T cells (middle), and multiparameter memory phenotype of T cells (bottom). (Fig 4 tab) MFI of IFNγ staining of CAR^+^ T cells following 6 hour co-culture with target cells. (Fig 5 tab) 4-hour chromium release assay of T cells co-cultured with target cells. (Fig 6 tab) BLI flux kinetics of Kasumi2-ffLuc-mKate cells following challenge with CAR^+^ T cells (top) and days of mouse euthanasia (bottom).
(XLSX)
